# Metagenomic profiles of *Dermacentor* tick pathogens from across Mongolia, using next generation sequencing

**DOI:** 10.3389/fmicb.2022.946631

**Published:** 2022-08-10

**Authors:** Doniddemberel Altantogtokh, Abigail A. Lilak, Ratree Takhampunya, Jira Sakolvaree, Nitima Chanarat, Graham Matulis, Betty Katherine Poole-Smith, Bazartseren Boldbaatar, Silas Davidson, Jeffrey Hertz, Buyandelger Bolorchimeg, Nyamdorj Tsogbadrakh, Jodi M. Fiorenzano, Erica J. Lindroth, Michael E. von Fricken

**Affiliations:** ^1^National Center for Zoonotic Diseases, Ulaanbaatar, Mongolia; ^2^Department of Global and Community Health, George Mason University, Fairfax, VA, United States; ^3^Department of Entomology, US Army Medical Directorate of the Armed Forces Research Institute of Medical Sciences (USAMD-AFRIMS), Bangkok, Thailand; ^4^School of Veterinary Medicine, Mongolian University of Life Sciences, Ulaanbaatar, Mongolia; ^5^Department of Chemistry and Life Science, US Military Academy, West Point, NY, United States; ^6^Naval Medical Research Unit TWO (NAMRU-2), Sembawang, Singapore

**Keywords:** next generation sequencing, *Dermacentor*, Mongolia, tick-bome disease, *Rickettsia*, *Bartonella*, *Anaplasma*, surveillance

## Abstract

Tick-borne diseases are a major public health concern in Mongolia. Nomadic pastoralists, which make up ~ 26% of Mongolia’s population, are at an increased risk of both tick bite exposure and economic loss associated with clinical disease in herds. This study sought to further characterize tick-borne pathogens present in *Dermacentor* ticks (*n* = 1,773) sampled in 2019 from 15 of Mongolia’s 21 aimags (provinces). The ticks were morphologically identified and sorted into 377 pools which were then screened using Next-Generation Sequencing paired with confirmatory PCR and DNA sequence analysis. *Rickettsia* spp. were detected in 88.33% of pools, while *Anaplasma* spp. and *Bartonella* spp. were detected in 3.18 and 0.79% of pools, respectively. Khentii had the highest infection rate for *Rickettsia* spp. (76.61%; CI: 34.65–94.79%), while Arkhangai had the highest infection rate for *Anaplasma* spp. (7.79%; CI:4.04–13.72%). The exclusive detection of *Anaplasma* spp. in tick pools collected from livestock supports previous work in this area that suggests livestock play a significant role in disease maintenance. The detection of *Anaplasma, Bartonella,* and *Rickettsia* demonstrates a heightened risk for infection throughout Mongolia, with this study, to our knowledge, documenting the first detection of *Bartonella melophagi* in ticks collected in Mongolia. Further research deploying NGS methods is needed to characterize tick-borne pathogens in other endemic tick species found in Mongolia, including *Hyalomma asiaticum* and *Ixodes persulcatus*.

## Introduction

Ticks and the pathogens they carry pose a significant threat to both human and animal health. This holds true in Mongolia, where an estimated 26% of the population continues to live a nomadic pastoral lifestyle and 37% of households own livestock (Odontsetseg et al., 2009; Boldbaatar et al., 2017; Barnes et al., 2020). These populations spend prolonged periods of time moving herds through tick habitats, resulting in a heightened risk for exposure to ticks and tick-borne diseases (TBDs). The Mongolian economy is also likely impacted by the effects of TBDs, where an estimated 67 million heads of livestock are present within the country^1^ and roughly 18% of the nation’s GDP comes from animal-related products (Odontsetseg et al., 2009). In neighboring China, an estimated $70 million every year is lost due to the impact of tick-borne disease impacts on small mammal production (Yin and Luo, 2007).

Ticks gathered in Mongolia have previously tested positive for various TBDs, including *Anaplasma* spp., *Borrelia* spp., Crimean-Congo hemorrhagic fever, *Ehrlichia* spp.*, Rickettsia* spp., and tick-borne encephalitis virus (Moore et al., 2018; Voorhees et al., 2018; Černý et al., 2019; von Fricken et al., 2020a). Rickettsial diseases are of particular concern due to high rates of severe illness and death in previously healthy individuals, (Aung et al., 2014; Biggs, 2016; von Fricken et al., 2018). A previous study by our team found that 20% of humans and livestock animals in Mongolia have had past exposure to *Rickettsia* spp., with variations observed by geographic location (von Fricken et al., 2018). This also held true when examining previous exposure to *Anaplasma* spp., which was detected in 37% of nomadic herders and over 40% of livestock (von Fricken et al., 2018). We also have detected *Anaplasma ovis* infection rates as high as 80% in sheep and 69% in goats, which aligns with what has previously been detected in ticks from the same region (Ochirkhuu et al., 2017; von Fricken et al., 2018, 2020a; Enkhtaivan et al., 2019; Fischer et al., 2020; Chaorattanakawee et al., 2022). Anaplasmosis in livestock can result in anoxia, abortions, infertility, significant weight loss, and even death, all of which can impact economic security in pastoralist communities.

*Dermacentor* ticks are the most common and one of the more important ticks of medical and veterinary concern within Mongolia due to their wide geographic range and the pathogens they carry (Černý et al., 2019). Ticks collected from southern and central aimags have previously had high pool positivity rates (> 80%) for *Rickettsia* spp., with molecular detections of *R. raoultii, R. sibirica mongolitimonae,* and *R. sibirica* reported (Fischer et al., 2020; von Fricken et al., 2020b). In contrast, a study of pathogens within ticks collected from aimags of central Mongolia found lower overall levels of *Anaplasma* spp. within *Dermacentor* ticks, although the infectivity rates increased substantially when specifically examining ticks removed from livestock (von Fricken et al., 2020a). Additional pathogens have been detected within *Dermacentor* ticks collected from Mongolian aimags include *Babesia caballi, B. equi, Borrelia afzelii, Candidatus* Midichloria sp., *Candidatus* Neoehrlichia mukurensis*, Theileria equi,* and *T. orientalis* (Battsetseg et al., 2001; Javkhlan et al., 2014; Fischer et al., 2020). In neighboring countries, pathogens reported from *Dermacentor* spp. ticks include *Babesia venatorum, Borrelia miyamotoi, Brucella* spp., *Francisella tularensis* subsp. *holarctica, Rickettsia aeschlimannii,* and the Far Eastern genotype of tick-borne encephalitis virus (Zhang et al., 2008; Wei et al., 2016; He et al., 2018; Yin et al., 2018; Huang et al., 2020; Gao et al., 2021; Jiao et al., 2021).

The potential threat tick-borne diseases present to both the Mongolian population and its growing ecotourism industry is substantial, given the high rates of various pathogens reported in previous tick survey studies (Černý et al., 2019; Fischer et al., 2020; von Fricken et al., 2020b). Improved molecular characterization of TBDs within Mongolia may help inform future preventative measures for locals and visitors, while also establishing baseline sequence data to monitor evolution over time. The variety of tick-borne pathogens found within Mongolia complicates attempts to fully characterize pathogens found in samples collected within the country. Our research group has recently used an analytical workflow on livestock blood samples from three aimags in Mongolia, initially applying next-generation sequencing (NGS) to obtain a snapshot of pathogen groups present, followed by conventional PCR and Sanger sequencing for confirmation and species characterization (Chaorattanakawee et al., 2022). In this study, we deploy Next-Generation Sequencing for on *Dermacentor* ticks collected from a wide geographic range of Mongolia to further our understanding of tick-borne pathogens in this region.

## Materials and methods

**Figure 1:**
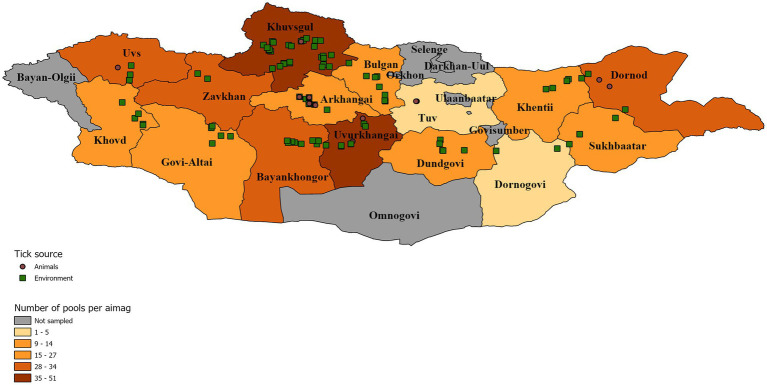
Distribution of tick collection events symbolized according to collection source. A map of Mongolia representing the location of tick pools that were chosen for further analysis. Tick pools are symbolized according to their collection source off animals (brown circle) or from the environment (green square). Individual aimags are colored according to the number of pools analyzed per aimag to demonstrate sampling intensity.

*Dermacentor* ticks were collected from the environment (questing) and off domestic animals from 15 aimags across Mongolia in 2019 (Uvs, Khovd, Govi-Altai, Zavhan, Khuvsgul, Arkhangai, Bayankhongor, Arkhangai, Uvurkhangai, Bulgan, Tuv, Dundgovi, Khentii, Dornogovi, Sukhbaatar, and Dornod; Figure 1). Adult ticks were morphologically identified as *D. nuttalli* or to the genus level as *Dermacentor* spp. by entomologists using local keys (Boldbaatar and Byambaa, 2015). In total, 7,275 ticks were collected and sorted into 1,489 pools according to location and collection source (environment vs. animal). Of these pools, 377 pools of adult stage ticks, representing pools from all sampled provinces, were selected for analysis by next-generation sequencing, including 51 pools collected from livestock (Tables 1, 2). Whole ticks in 250 μl of ATL buffer were punctured with a fine tip under a stereomicroscope to release the tissue from the hard chitin exoskeleton prior to adding 2 mg/ml of Proteinase K solution. Samples were then incubated at 55°C overnight. A total volume of 250 μl homogenized solution was then used for DNA extraction on the QIAsymphony® SP instrument with QIAsymphony® DSP DNA Mini Kit using Tissue LC 200 DSP protocol (Qiagen, Hombrechtikon, Switzerland). The DNA was eluted in 50 μl of ATE buffer and stored at −20°C until use.

**Table 1:** Samples selected for pathogen screening by next-generation sequencing (NGS) in this study.

Provinces	Number of tick pools	Number of ticks	Number of tick pools selected for NGS	Number of ticks in pools selected for NGS
Arkhangai	61	305	27	135
Bayankhongor	101	496	29	143
Bulgan	14	56	14	56
Dornod	122	599	34	159
Dornogovi	5	18	5	18
Dundgovi	47	226	27	126
Govi-Altai	135	675	24	120
Khentii	8	17	8	17
Khovd	162	810	26	130
Khuvsgul	223	1,050	51	232
Sukhbaatar	140	693	27	133
Tuv	4	21	4	21
Uvs	162	810	29	145
Uvurkhangai	124	594	42	188
Zavhan	181	905	30	150
Total	1,489	7,275	377	1773

**Table 2:** Pool positivity rate by collection source [% and (95% CI)].

Pathogens	Sources (% Infection, 95% CI)			
	Animals (*N* = 51)	Environment (*N* = 307)	Rock and bush (*N* = 19)	Total
*Rickettsia*	41 [80.4% (69.5, 91.3%)]	275 [89.6% (86.2, 93.0%)]	17 [89.5% (75.7, 103.0%)]	333
*Anaplasma*	12 [23.5% (11.9, 35.2%)]	0	0	12
*Bartonella*	1 [1.96% (−1.8, 5.8%)]	2 [0.7% (−0.3, 1.6%)]	0	3

### Bacterial 16S DNA amplification

Nested PCR was performed as described in Chaorattanakawee et al., 2022 to amplify both the V1-V6 region and V3-V4 region of the bacterial 16S rDNA. Each round of PCR included both an ultrapure DNA/RNA-free water negative control and a mock DNA extraction control. The nested PCR amplicon products were isolated using AMPure XP magnetic beads and the quality of the products was assessed as previously described (Chaorattanakawee et al., 2022). Amplicon products were stored at −20°C until further analysis.

### Library preparation and sequencing

The Nextera XT Index Kit v2 (Illumina) was used for index PCR to attach the dual indices and Illumina sequencing adapters to purified 16S amplicons as previously described. Each batch of indexing reactions included a DNA/RNA-free water as a negative control. The index PCR products were cleaned using AMPure XP beads, followed by library purity analysis using the QIAxcel Advanced System (Qiagen). The index libraries were then quantified using the Qubit dsDNA HS Assay Kit (Invitrogen). Libraries were denatured with NaOH according to the manufacturer’s protocol (Ilumina). Sequencing was performed using the MiSeq Reagent Kit V3 with the Ilumina Miseq System. A 10% PhiX internal control (Ilumina) was included in each low-diversity library run.

### NGS data analysis

Sequence reads produced by the Ilumina MiSeq system were processed using the CLC Genomics workbench (v 11.0.1) and CLC microbial genomics module (v 3.0; Qiagen, Aarhus A/S1), which included merging paired reads, primer sequence removal, low read sample removal, and chimeric sequence removal. The filtered sequences were then clustered into operational taxonomic unites (OTUs) using a threshold of 97% sequence identity and the reference OTU database downloaded from the Greengenes database (v 13.8) and SILVA 16S (v 132). Pathogen reads detected in the negative controls represented cross-contamination and were used to subtract respective reads detected in samples.

### Pathogen characterization by PCR and sanger sequencing

To confirm the detection of pathogens and the taxonomic assignment as indicated by NGS analysis, PCR and DNA sequencing were conducted on NGS samples with read counts above a set threshold. The assays and gene targets for selected pathogens (*Anaplasma, Bartonella, Rickettsia, Coxiella*) were detailed previously in Takhampunya et al. (2019). PCR amplification products were cleaned using the ExoSAP-IT kit (Applied Biosystem), followed by cycle-sequencing and sequencing using the SeqStudio Genetic Analyzer (Applied Biosystems), as previously described (Chaorattanakawee et al., 2022). Sample sequences were assembled using Sequencher (v 5.1, Gene Codes Corp.) and aligned with GenBank reference sequences using the MUSCLE codon alignment program. Maximum likelihood phylogenetic trees were constructed for each bacterial target gene using MEGA 6.

### Mapping

ArcGIS Pro (v 2.8.0, ESRI) was used for spatial visualizations of data, including tick collection events, tick collection source, and pathogen detection. The map layer of Mongolia and its delineated aimags was accessed from ESRI.^2^

### Statistical analysis

To estimate the probability of pathogen detection within the pooled samples, prevalence rates, maximum likelihood estimates (MLE) and minimum infection rates (MIR) were calculated, which is standard when analyzing pooled tick data. The MLE and MIR estimates were conducted in Excel with the use of the CDC’s Mosquito Surveillance Software tool which calculate point and confidence intervals using pooled data that take into account individual pool sample sizes to estimate infection rates.^3^

## Results

### Detection of *Rickettsia*, *Anaplasma*, and *Bartonella*

**Figure 2:**
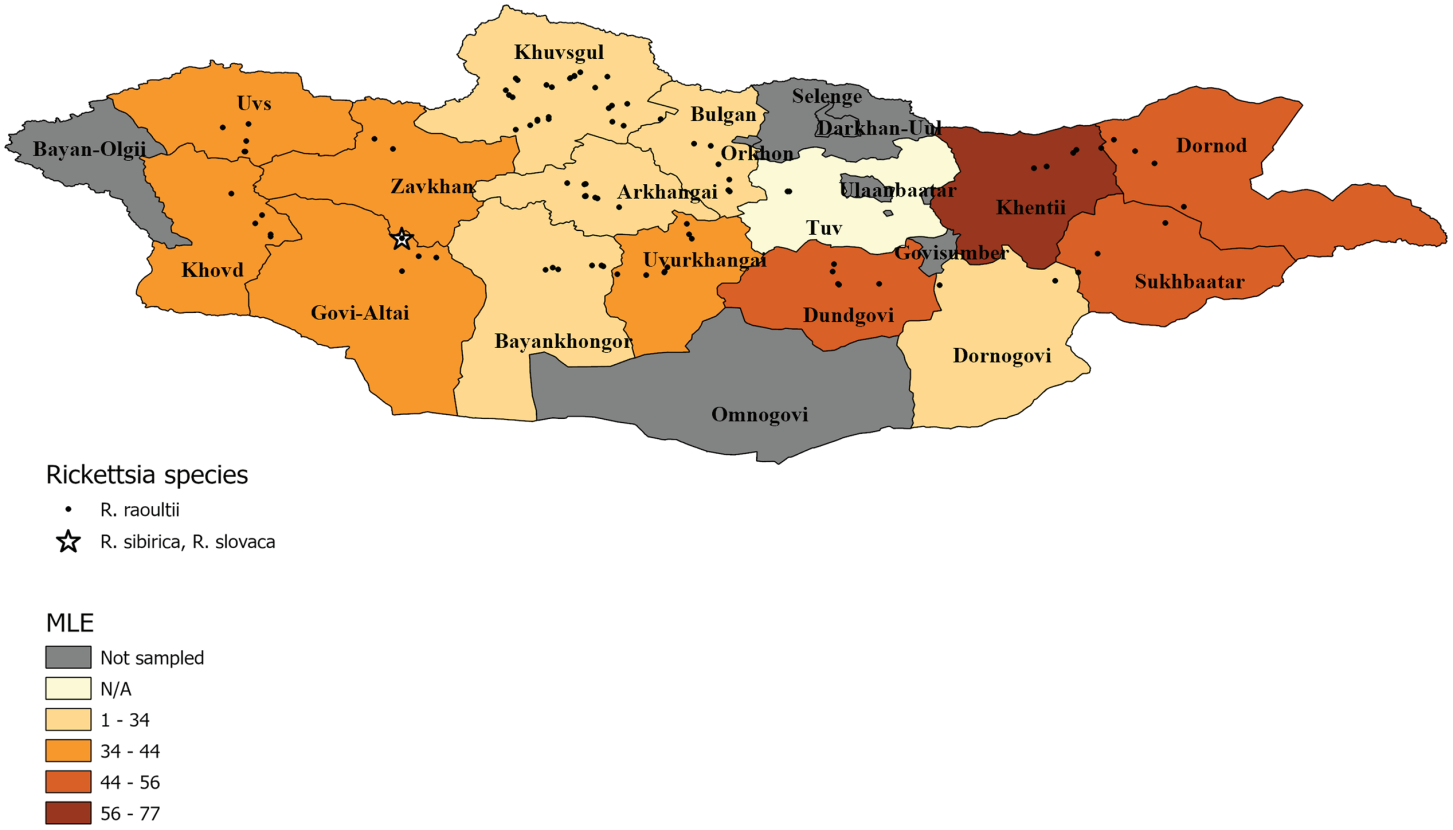
Pool MLE for *Rickettsia* spp. A map showing the distribution of identified *Rickettsia* species, with an aimag color gradient representing the *Rickettsia* spp. MLE of sampled pools within the aimag. MLE calculation for Tuv is N/A because detection rate was 100%.

The summary results for the *Rickettsia* spp., confirmed through qPCR analysis and DNA sequencing, are presented in Table 3 and Figure 2 Overall, *Rickettsia* spp. were detected in tick pools from all aimags sampled, with 88% of pools testing positive (333/377). The highest *Rickettsia* spp. pool detection rate was seen in Tuv (100%) followed by: Dornod (97%) Dundgovi (96%) and Sukhbaatar (96%), while the Bulgan aimag showed the lowest pool positivity rate (57%). Maximum likelihood estimates (MLE) found an average prevalence of 37.30% (95%CI: 33.50–41.01%), where Dornod aimag had the highest MLE of 55.40% (95%CI: 34.38–70.67%) and a MIR of 20.75% (95%CI: 14.45–27.06%) and Bulgan had the lowest MLE of 18.34% (95%CI: 9.21–30.23%) with a MIR of 14.29% (95%CI: 5.12–23.45%). In general, higher MLEs were found in tick pools collected from eastern and western aimags of Mongolia, with lower MLEs seen in central aimags (Figure 2).

**Table 3:** Maximum likelihood estimates of *Rickettsia* spp. by region based on confirmatory results including 95% confidence intervals.

*Rickettsia* spp.					
Province	Positive pools	Total number of ticks	MLE		
			Point	Low	High
Arkhangai	20/27 (74%)	135	23.7	14.9	33.3
Bayankhongor	24/29 (83%)	143	31.2	20.0	42.4
Bulgan	8/14 (57%)	56	18.3	9.2	30.2
Dornod	33/34 (97%)	159	55.4	34.4	70.7
Dornogovi	4/5 (80%)	18	33.4	12.1	54.9
Dundgovi	26/27 (96%)	126	51.5	31.1	67.1
Govi-Altai	22/24 (92%)	120	39.2	23.7	52.9
Khentii	7/8 (88%)	17	76.6	34.7	94.8
Khovd	24/26 (92%)	130	40.1	24.7	53.7
Khuvsgul	41/51 (80%)	232	31	22.6	39.4
Sukhbaatar	26/27 (96%)	133	48.7	29.2	63.9
Tuv	4/4 (100%)	21	N/A	N/A	N/A
Uvs	27/29 (93%)	145	41.4	26.2	54.7
Uvurkhangai	39/42 (93%)	188	43.9	31	55.1
Zavhan	28/30 (93%)	150	41.8	26.6	55.0
Total	333/377 (88%)	1773	37.3	33.5	41.0

Summary results for the *Anaplasma* spp., confirmed through PCR and DNA sequencing of the tick pools, are presented in Table 4. Pools were found to have an overall positivity rate of 3.18% for *Anaplasma* spp. (12/377), with only ticks sampled from Arkhangai (33% of pools), Uvs (7% of pools) and Uvurkhangai (2% of pools) testing positive. MLE found an average prevalence of 0.69% (95%CI: 0.39–1.19%), with Arkhangai having the highest MLE of 7.79% (95%CI: 4.04–13.72%) and MIR of 6.67% (95%CI: 2.46–10.87%). In contrast, Uvurkhangai had an MLE of 0.53% (95%CI: 0.09–2.87%) with a MIR of 0.53% (95%CI: 0–1.57%).

**Table 4:** Maximum likelihood estimates of *Anaplasma* spp. by region based on confirmatory results including 95% confidence intervals.

*Anaplasma* spp.					
Province	Positive pools	Total number of ticks	MLE		
			Point	Low	High
Arkhangai	9/27 (33%)	135	7.8	4.0	13.7
Bayankhongor	0/29 (0%)	143	–	–	–
Bulgan	0/14 (0%)	56	–	–	–
Dornod	0/34 (0%)	159	–	–	–
Dornogovi	0/5 (0%)	18	–	–	–
Dundgovi	0/27 (0%)	126	–	–	–
Govi-Altai	0/24 (0%)	120	–	–	–
Khentii	0/8 (0%)	17	–	–	–
Khovd	0/26 (0%)	130	–	–	–
Khuvsgul	0/51 (0%)	232	–	–	–
Sukhbaatar	0/27 (0%)	133	–	–	–
Tuv	0/4 (0%)	21	–	–	–
Uvs	2/29 (7%)	145	1.4	0.4	4.8
Uvurkhangai	1/42 (2%)	188	0.5	0.1	2.9
Zavhan	0/30 (0%)	150	–	–	–
Total	12/377 (3%)	1773	0.7	0.4	1.2

Table 5 summarizes the results for the *Bartonella* spp., confirmed through PCR and DNA sequencing of the tick pools. The overall *Bartonella* spp. pool positivity rate was found to be 0.79% (3/377), with Arkhangai being the only region with positive pools (3/27 pools). Maximum likelihood estimates (MLE) found an overall prevalence of 0.17% (95% CI: 0–0.06–0.50%). Arkhangai had a MLE of 2.33% (95%CI: 0.78–6.37%) and a MIR of 2.22% (95%CI: 0–4.71%). A full list of sequence accession numbers by gene target and microorganism can be found in Table 6.

**Table 5:** Maximum likelihood estimates of *Bartonella* spp. by region based on confirmatory results including 95% confidence intervals.

*Bartonella* spp.					
Province	Positive pools	Total number of ticks	MLE		
			Point	Low	High
Arkhangai	3/27 (11%)	135	2.3	0.8	6.4
Bayankhongor	0/29 (0%)	143	–	–	–
Bulgan	0/14 (0%)	56	–	–	–
Dornod	0/34 (0%)	159	–	–	–
Dornogovi	0/5 (0%)	18	–	–	–
Dundgovi	0/27 (0%)	126	–	–	–
Govi-Altai	0/24 (0%)	120	–	–	–
Khentii	0/8 (0%)	17	–	–	–
Khovd	0/26 (0%)	130	–	–	–
Khuvsgul	0/51 (0%)	232	–	–	–
Sukhbaatar	0/27 (0%)	133	–	–	–
Tuv	0/4 (0%)	21	–	–	–
Uvs	0/29 (0%)	145	–	–	–
Uvurkhangai	0/42 (0%)	188	–	–	–
Zavhan	0/30 (0%)	150	–	–	–
Total	3/377 (0.8%)	1773	0.2	0.0	0.5

**Table 6:** Identity and Genbank accession numbers for *Anaplasma*, *Bartonella* and *Rickettsia* spp. from pooled samples *Dermacentor* spp.

Target Gene	Organism	Location	GenBank#	Identity
*gltA*	*Bartonella schoenbuchii*	Arkhangai	OM281134-OM281135	99.21% AJ564635.1
	*B. melophagi*	Arkhangai	OM281136	100% AY692475.1
	*Rickettsia raoultii*	Arkhangai, Bayankhongor, Dornogovi, Dornod, Dundgovi, Govi-Altai, Khentii, Khovd, Khuvsgul, Sukhbaatar, Tuv, Uvs, Uvurkhangai, Zavhan	OM28112; OM281162; OM281137-OM281146; OM281148-OM281155; OM281157; OM281160-OM281168; OM281170-OM281171; OM2811173-OM281177; OM281179-OM281185; OM281187-OM281192; KU961538;	100% MT178337.1
	*R. raoultii*	Dornod, Khovd, Khuvsgul, Uvs	OM281156; OM281158; OM281159; OM281169; OM281178; OM281186	100% OK638145.1
	*R. sibirica/R. slovaca*	Govi-Altai	OM281147	100% MG811709.1;
*ompA*	*R. raoultii*	Bayankhongor, Dornod, Govi-Altai, Khentii, Khovd, Khuvsgul, Sukhbaatar, Uvs, Uvurkhangai	OM281193-OM281217	100% MK726326.1
16S rRNA	*Anaplasma ovis* *A. capra* *A. centrale* *A. marginale*	Arkhangai	OM320148-OM320155	100% MN266936.1
	*A. ovis*	Uvs	OM320157	100% MN266936.1
*groEL*	*A. ovis*	Arkhangai, Uvs	OM281118-OM281120; OM281122-OM281128	99.69% MT268377.1
	*A. ovis, A. centrale, A. marginale*	Arkhangai	OM28121	99.39% MT268375.1; 92.05 KY305559.1
	*A. ovis*	Uvs	OM281232	100% MH292916.1
	*A. ovis*	Arkhangai	OM281229-OM281231	100% MH292916.1
16S rRNA	Coxiella endosymbiont of *Dermacentor marginatus*	Arkhangai, Dornod, Dornogovi, Khentii, Khuvsgul, Sukhbaatar, Uvurkhangai	OM333168-OM333184	99.44% MZ047981.1

### Pathogen detection by tick source

The pathogen pool positivity rate by tick collection source is detailed in Table 2. *Rickettsia* spp. was detected in 80.4% (95% CI 69.5, 91.3) of tick pools removed from livestock animals, with Tuv having the highest pool positivity rate (100%) and Khuvsgul having the lowest pool positivity rate (33.3%; Table 3). The *Rickettsia* spp. infection rate in ticks collected from different sources (animal vs. environment including from rock and bush) was compared using Chi-square test and no significant difference was found (Chi-square = 2.7685, *df* = 1, *value of p* = 0.09614). Of note, *Anaplasma* spp. was only detected in tick pools collected from animals, with a pool positivity rate of 23.5% (95% CI 11.9, 35.2). Arkhangai had the highest level of pool positivity, with 47.4% of tick pools collected from animals having *Anaplasma* spp. DNA present (Table 1). *Bartonella* spp. was detected in 1.96% (95% CI -1.8, 5.8) of pools of ticks removed from animals and 0.7% (95% CI –0.3, 1.6) of tick pools collected from the environment. As discussed above, all three pools testing positive for *Bartonella* spp. came from the Arkhangai aimag, with one pool representing ticks collected from animals (5.26% of animal tick pools from Arkhangai (Table 2), and the other two pools being ticks collected from the environment (25% of environmental tick pools from Arkhangai).

### Pathogen species confirmation

**Figure 3:**
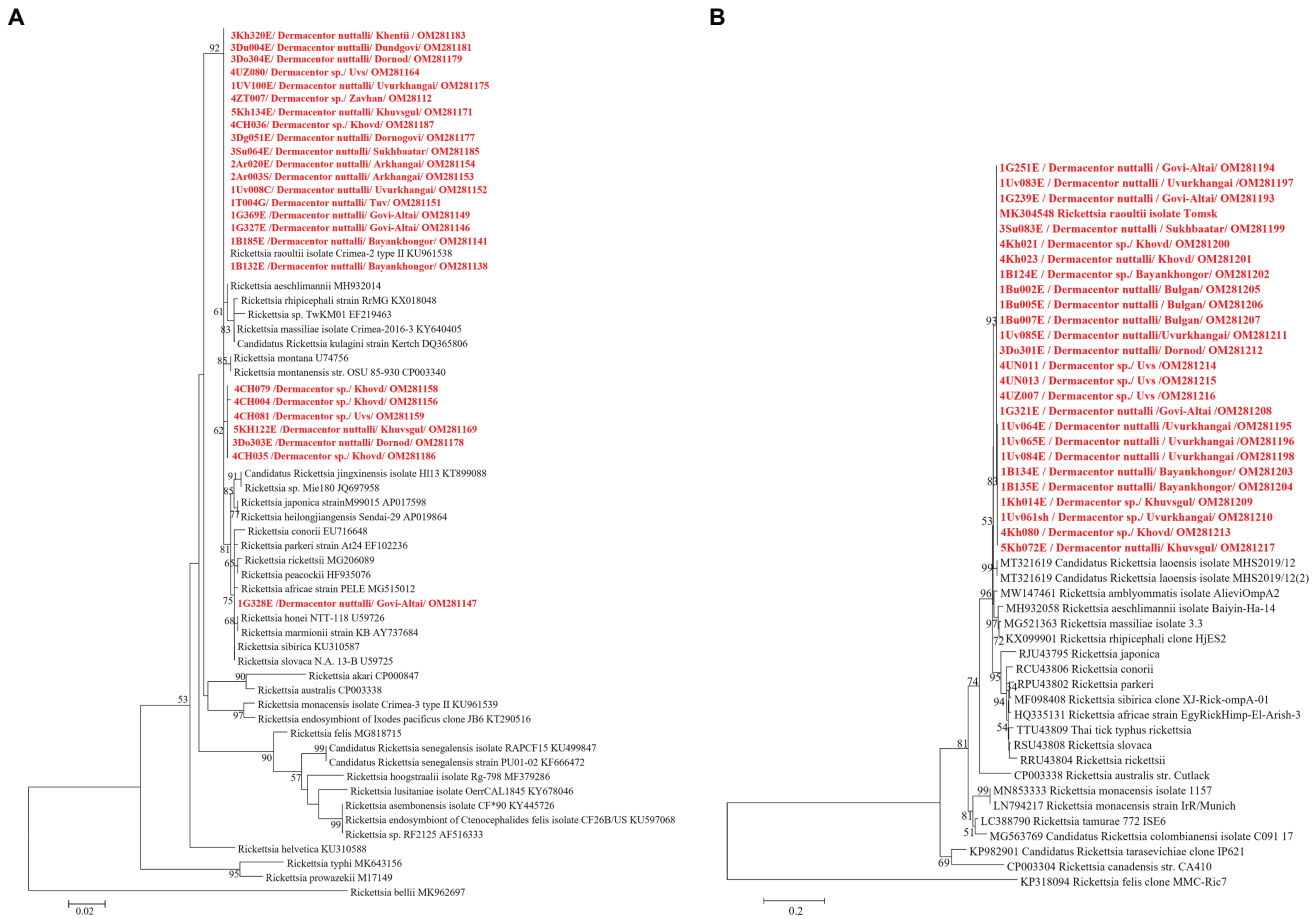
Maximum likelihood (ML) tree was constructed from *glt*A gene **(A)** and *omp*A gene **(B)** of *Rickettsia* spp. using T92 + G model with 1,000 bootstrap replicates (> 50% are shown on each node). Sequences of tick samples in this study are shown in red letters.

**Figure 4:**
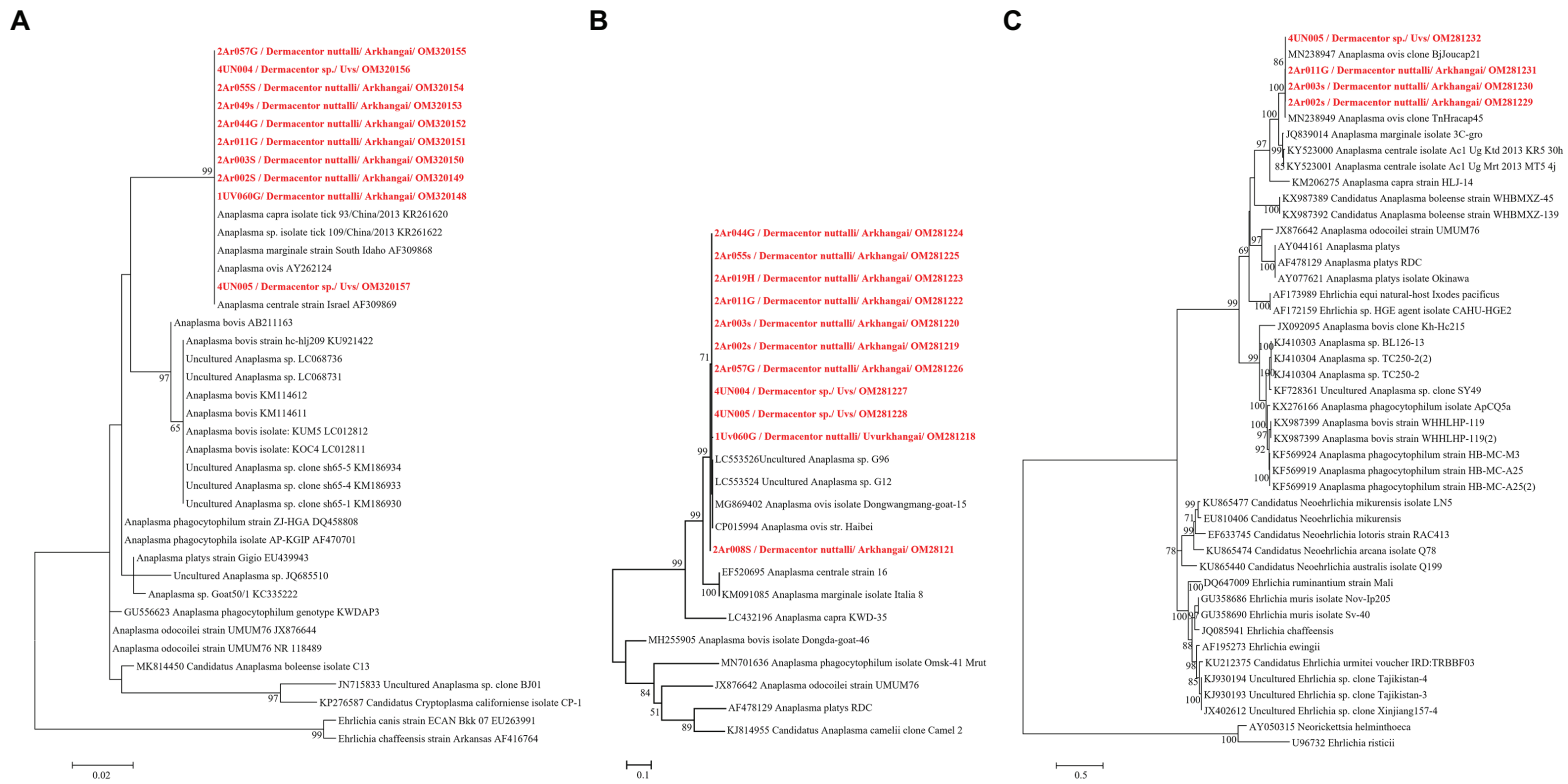
ML tree was constructed from 16S rRNA **(A)** and *groEL* genes using K9 + G **(B)** and TN93 + G + I **(C)** models, respectively, with 1,000 bootstrap and value over 50% are indicated on each node.

**Figure 5:**
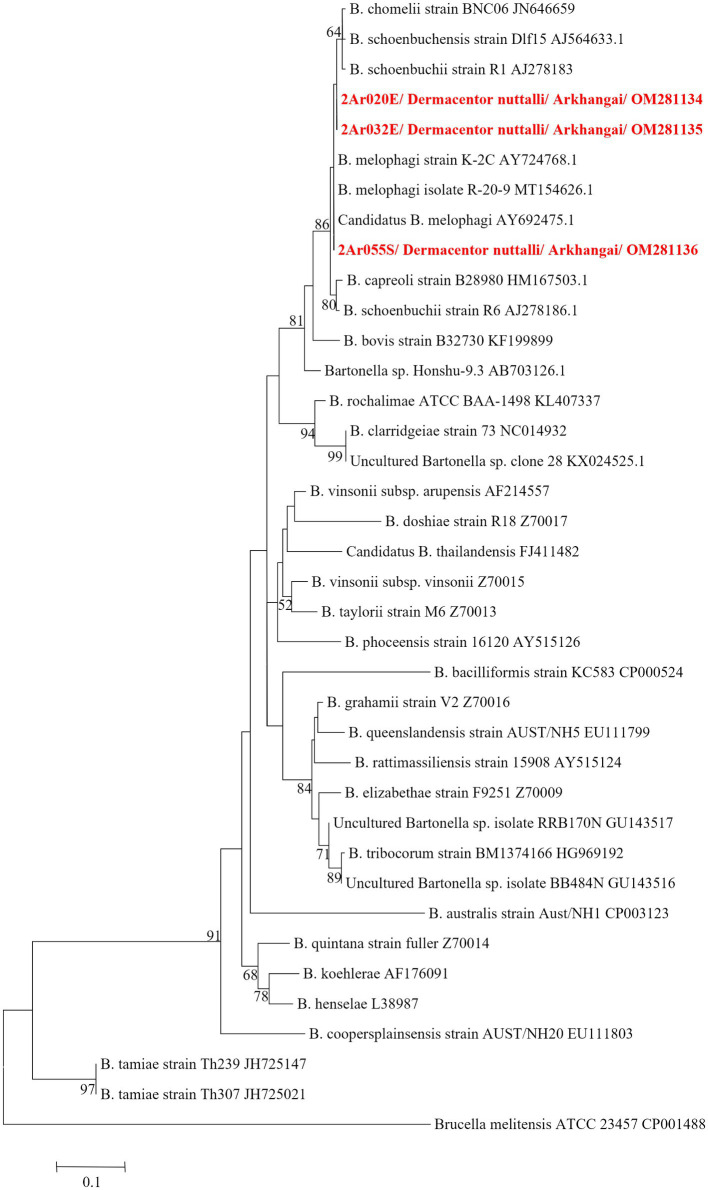
ML tree constructed from *glt*A gene of *Bartonella* spp. using T92 + G model with 1,000 bootstrap replicates (> 50% value are shown on each node). Sequence of tick samples in this study are indicated in red letters.

**Figure 6:**
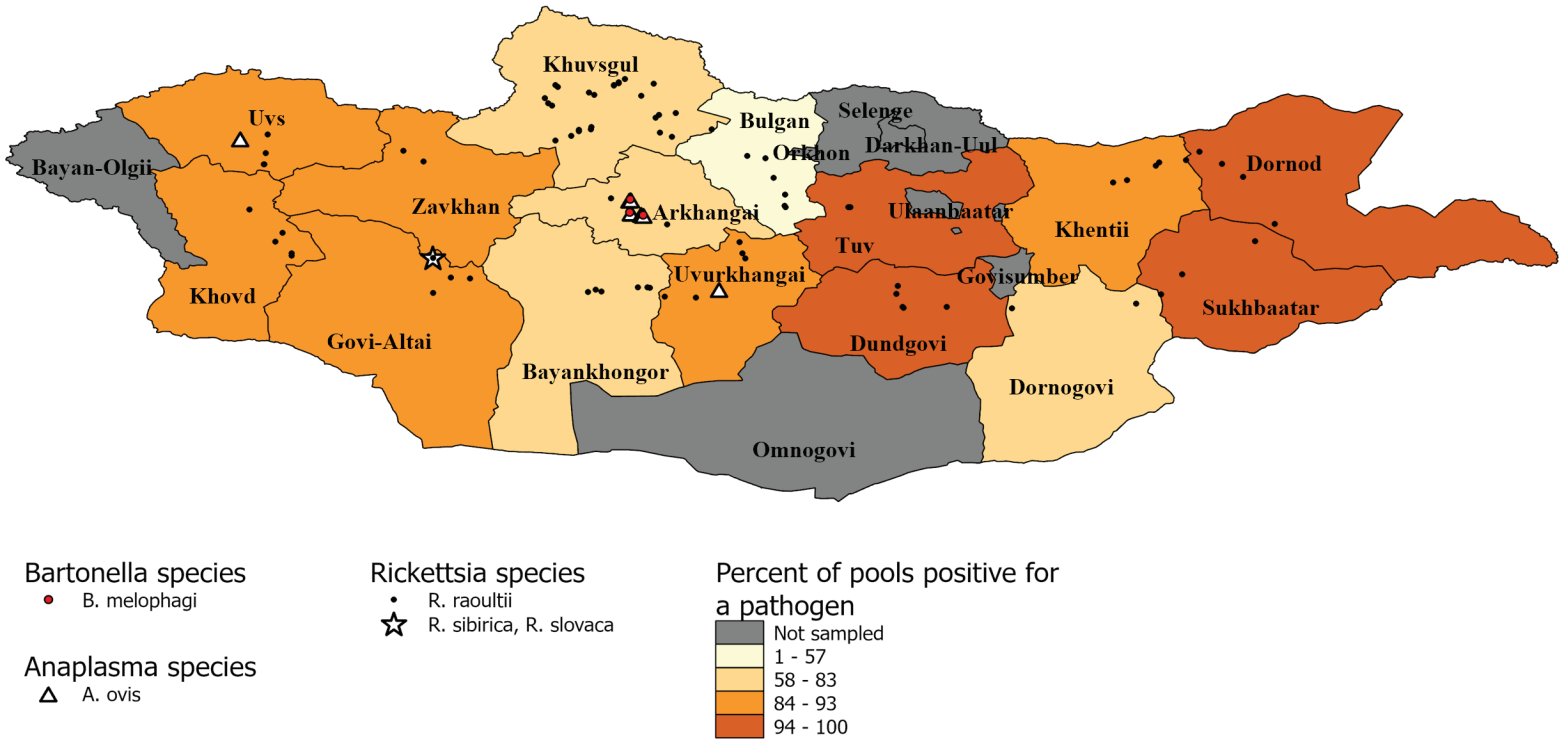
Distribution of identified microbial species and pool positivity rate by aimag. A map demonstrating the geographic distribution of microbial species identified within the sampled tick pools. Each identified microbial species has a different symbol. The aimags are colored according to the proportion of pools that tested positive for *Anaplasma, Bartonella,* or *Rickettsia* species.

DNA sequencing allowed for pathogen species confirmation of pools testing positive for the various bacterial groups. Within *Rickettsia* spp. positive tick pools, the *gltA* and *omp*A sequences were analyzed, with the summarizing maximum likelihood (ML) tree presented in Figure 3. As shown in the ML tree, most pools were identified as having *R. raoultii* (n = 332), although one environmental tick pool from Govi-Altai had a pathogen identified as *R. sibirica*/*Rickettsia slovaca* (100% sequence identity). *Anaplasma* species were identified by analyses of both the 16S rDNA and *groEL*, placing *Anaplasma*-positive pools within the *A. capra, A. centrale, A. marginale*, and *A. ovis* group, with all *Anaplasma-*positive pools eventually being grouped within the *A. ovis* group (*n* = 12, Figure 4). Finally, ML *gltA* gene analysis of the three pools that tested positive for *Bartonella* spp. identified the species as *Bartonella melophagi* (Figure 5). Figure 6 summarizes the geographic distribution of identified microbial species as well as the proportion of pools within an aimag that tested positive for *Anaplasma, Bartonella,* or *Rickettsia* species. A higher proportion of tick pools tested positive for a pathogen in the eastern and western part of Mongolia, which was largely driven by high detection rates of *R. raoultii*.

## Discussion

This study continues previous work describing the microbial diversity found within *Dermacentor* ticks of Mongolia, applying next-generation sequencing to ticks collected from a wider geographic range. Findings from this study reiterate that *Rickettsia* spp., specifically *R. raoultii*, are highly prevalent across Mongolia, with aimag pools having a positivity rate of above 50%. While previous work has documented high pool positivity levels for *Rickettsia* spp. in *Dermacentor* ticks from southern and central aimags of Mongolia (Fischer et al., 2020; von Fricken et al., 2020b), this study has a much wider geographic range and represents, to our knowledge, the first time NGS methods have been applied to testing *Dermacentor* ticks in Mongolia. The finding of higher MLEs and pool-positivity rates in the eastern part of Mongolia may indicate a potential hotspot for *Dermacentor* tick-related *Rickettsia* spp. exposure, warranting future human and animal serological studies in these areas. Of note, the total MLE in this study (37.30%; 95% CI: 33.50–41.01) is similar to a previous work that sampled ticks from five southern aimags, where the MLE for *Dermacentor* spp. was 33.2% (95% CI: 30.1–36.2; von Fricken et al., 2020b). Additionally, zero larvae and few nymphs were found across 191 geographically distinct collection events spread out through 15 aimags, which we believe is suggestive of *Dermacentor* ticks spending earlier life cycle stages underground in rodent burrows, given harsh dry winter seasons common in Mongolia. This theory is also supported by high levels of *Rickettsia* detection in rodent reservoirs, where 17/18 *Meriones unguiculatus* (Mongolian Gerbil) tested positive for *Rickettsia* DNA, with this rodent commonly found across Mongolia (Pulscher et al., 2018). When paired with evidence of transovarial transmission of *Rickettsia* in *Dermacentor* ticks from Mongolia (Moore et al., 2018), it is not surprising to observe such high detection rates across this wide geographic range. The *Rickettsia* species identified through NGS analysis include *R. raoultii* and *R. sibirica/R. slovaca*, which aligns with previous reports of *R. raoultii* and *R. sibirica* from *Dermacentor* spp. collected from the Omnogovi, Dornogovi, Govi-Altai, Khovd, Khentii and Bayankhongor aimags (Fischer et al., 2020; von Fricken et al., 2020b). Infections with *R. raoultii* typically manifest with eschars and lymphadenopathy, although severe cases have been reported with pulmonary edema (Li et al., 2018). Similarly, *R. sibirica* subspecies present with non-specific flu-like symptoms accompanied by a rash and eschar, with more severe complications such as disseminated intravascular coagulation, renal failure, and neurological symptoms (Nouchi et al., 2018). Therefore, the detection of these pathogens across a wide geographic distribution of ticks within Mongolia represent a major public health threat that is likely under reported in pastoral communities, due to limited access to healthcare in rural regions and low treatment-seeking behaviors within this population (Lkhagvatseren et al., 2019).

While only seen in three aimags (Arkhangai, Uvs, and Uvurkhangai), *Anaplasma* spp. was still detected in 3.18% of pools overall, eventually being identified as *A. ovis*. This *Anaplasma* species causes anaplasmosis in sheep, goats, and wildlife ruminants, often characterized as a subclinical disease which can lead to reduced milk production and spontaneous abortions (Cabezas-Cruz et al., 2019). Of note, all positive pools for *Anaplasma* spp. came from ticks removed from livestock, primarily sheep and goat, with zero detections occurring in environmental samples. A previous study of *Anaplasma* spp. and *Ehrlichia* spp. within ticks collected from central Mongolia reported a similar pattern of high MLE rates when ticks collected from animals were considered separately from ticks collected from the environment (Shao et al., 2020; von Fricken et al., 2020a). This pattern of pathogen distribution within ticks may result from ticks taking partial blood meals from infected livestock hosts and then detaching and reattaching to other livestock hosts, spreading the disease within herds in the process. We do not believe rodents or transovarial transmission plays a significant role in *A. ovis* transmission cycles given the absence of detection in such a large sample. These findings highlight an important component of *Anaplasma* spp. disease ecology where livestock act as amplifying hosts. Given these observed patterns of *Anaplasma* spp. transmission within Mongolia, the lack of *Anaplasma* spp. detection in many of the aimags in this study should not be interpreted as an actual absence of this microbe group, as many of the pools within these *Anaplasma*-negative aimags were primarily pools collected from the environment. Further investigation into co-feeding transmission between ticks and potential vectors is warranted, when paired with the high seroprevalence and pathogen detection found in previous studies screening livestock (Zhang et al., 2008; Jiao et al., 2021).

*Bartonella* spp. are typically transmitted by fleas and lice, however there is an ongoing larger discussion about what potential role ticks play within transmission cycles (Angelakis et al., 2010; Cheslock and Embers, 2019). Studies that have suggested the possible role of ticks as vectors of *Bartonella* spp. include the reported presence of *Bartonella* spp. DNA within various tick groups collected from around the world, including *Dermacentor* ticks, and epidemiological studies have often noted tick bites preceding *Bartonella* spp.-related illnesses (Wikswo et al., 2007; Angelakis et al., 2010; Zając et al., 2015). Experimental data supporting tick-mediated transmission of *Bartonella* spp. is limited, but includes the ability of *Bartonella* spp. to replicate within multiple tick species cell lines (Billeter et al., 2009), the ability of *Bartonella-*infected ticks to transmit *Bartonella* infection between animal models while feeding (Noguchi, 1926; Reis et al., 2011), and the detection of *Bartonella* spp. DNA or bacilli in infected tick midgut, salivary glands, and feces (Cotté et al., 2008; Reis et al., 2011; Wechtaisong et al., 2021). Despite these findings, there is still a lack of consensus regarding the ability of ticks to vector *Bartonella* species. Regardless of whether ticks play a role in transmitting *Bartonella* in Mongolia, here we provide further evidence that *Bartonella melophagi* is present in Mongolia. Within this study, *Bartonella melophagi* was found in 11.1% of tick pools from the Arkhangai aimag, including a tick pool collected from sheep. Sheep are considered the reservoir host for *B. melophagi,* with sheep keds being the common insect vector of this pathogen (Maggi et al., 2009). Human infections with this microbe have been reported, resulting in flu-like symptoms, bite-site lesion, neurological symptoms, and heart irregularities (Maggi et al., 2009). Although our group has previously reported *B. melophagi* within Mongolian sheep, to our knowledge, this is the first report of *B. melophagi* within ticks collected from Mongolia (Chaorattanakawee et al., 2022). Of note, this *Bartonella* species has been reported in *Dermacentor, Hyalomma,* and *Rhipicephalus* ticks collected from Xinjiang, China (Ni et al., 2021). The detection of *B. melophagi* in both domestic animals and ticks of Mongolia emphasizes a need for future studies to characterize the disease ecology of this pathogen, including determining the role of ticks in disease transmission and the possibility of transboundary disease movement between Mongolia and China.

All tick pools analyzed by qPCR and conventional PCR targeting the *Coxiella burnetii* transposase gene were negative, suggesting the absence of this pathogen from the ticks sampled. Of note, 35.8% of pools tested positive for a *Coxiella*-like bacteria, warranting further investigation.

This study illustrates the utility of NGS in characterizing the diversity of tick-borne pathogens found in *Dermacentor* ticks collected from geographically distinct locations. We applied NGS to receive a “snapshot” of the various bacterial groups present within tick pools, which then guided confirmatory assays to allow for accurate identification of tick-borne pathogen species. Given the large number of tick-borne pathogens present within Mongolia, the use of a nontargeting analytical method is appropriate to avoid unintentionally excluding the detection of certain microbial species which might be missed relying on other detection processes. Importantly, certain results of this study corroborate what has previously been reported concerning the epidemiology of tick-borne pathogens within Mongolia. This includes the high prevalence of *Rickettsia* spp., particularly *R. raoultii*, among *Dermacentor* ticks collected across the country (Fischer et al., 2020; von Fricken et al., 2020b). We also report the detection of *A. ovis* exclusively in ticks removed from livestock, which is in agreement with a previous study demonstrating higher levels of *Anaplasma* spp. in ticks collected from animals (Shao et al., 2020; von Fricken et al., 2020a). While this study expands on the knowledge concerning the geographical distribution of *Rickettsia* spp. and *Anaplasma* spp. within *Dermacentor* ticks, the observation that many of the results are in agreement with previous studies indicates that NGS offers a valid, novel approach for the characterization of tick-borne pathogens. Of note, the novel detection of *Bartonella* spp. DNA within *Dermacentor* spp. ticks collected within Mongolia also demonstrates the ability of NGS to discover new pathogen-vector relationships, which may be more difficult to detect using other molecular processes. Future use of NGS to describe the microbial diversity found in the various tick species from Mongolia will further contribute to a more complete characterization of the tick-borne pathogens in circulation within the country. The results from this study will contribute to more detailed risk mapping for tick-borne pathogens, which will help inform disease prevention interventions that benefit populations at increased risk of disease exposure. The abilities of NGS to identify novel vector-pathogen associations will also prove to be vital for local health care practitioners by informing them on the various tick-borne diseases they should include as part of differential diagnoses strategies.

The use of NGS in epidemiological surveys of vector species such as ticks has many advantages over pathogen detection methods that are typically used in such studies, such as PCR and immunofluorescence. While this study focused primarily on clinically relevant pathogens, sequencing data resulting from NGS allows for the creation of a library of microbial sequences, which can promote the tracking of microbial evolution overtime (Deurenberg et al., 2017). Importantly, by surpassing the need for selecting pathogen-specific molecular probes, the use of NGS streamlines the ability of groups to rapidly identify uncommon or previously uncharacterized microbial agents, which may represent emergent diseases (Wu et al., 2021).

### Limitations

In this study, ticks were only identified morphologically, which limits our ability to infer findings beyond the genus level. The reliance on morphological identification of ticks may have led to misidentification of the tick species analyzed in this study, hence the decision to keep most of our discussion at the genus level. The decision to pool ticks within this study also introduces some limitations, including difficulties in determining the true prevalence of the various microbial agents that were detected. For example, in instances in which 100% of tick pools are positive, it is not possible to calculate a maximum likelihood estimate, which is what occurred for the Tuv aimag pools for our *Rickettsia* results. Although there was one pool in which all three pathogens were detected and 12 pools in which two pathogens were detected, discussion of co-infection status of ticks is also complicated by pooling of ticks. While the use of NGS may prove useful for the characterization of pathogenic microbes within ticks and other vector insect species, it is important to note that the detection of microbial DNA does not necessarily indicate the presence of viable microbial organisms within the tick sample. The detection of microbial DNA may also represent remnant DNA from a recent bloodmeal (Tokarz et al., 2019). Therefore, caution must be taken when interpreting NGS results from blood-feeding arthropods.

## Conclusion

Here we report the use of NGS to assess the diversity of pathogens within *Dermacentor* ticks collected from 15 different aimags of Mongolia. The results of this study highlight a high level of *Rickettsia* detected across all sampled aimags, including the presence of *R. sibirica/R. slovaca* in Govi-Altai, as well as detections of *A. ovis* in samples removed from livestock. These findings also highlight the first reported detection of *B. melophagi* in ticks from this region. Future studies should make use of NGS analysis to further characterize the diversity of pathogens found in other medically relevant tick species within Mongolia, as this method allows for the detection of multiple pathogens simultaneously.

## Author’s note

The material in this manuscript has been reviewed by the Walter Reed Army Institute of Research. There is no objection to its presentation and/or publication. The opinions or assertions contained herein are the private views of the author, and are not to be construed as official, or as reflecting true views of the Department of the Army, Department of the Navy, the Department of Defense, or the US Government. Multiple authors are military service members and/or federal/contracted employees of the United States government. The work prepared by DoD personnel in this manuscript was prepared as part of their official duties. Title 17 U.S.C. 105 provides that `copyright protection under this title is not available for any work of the United States Government.’ Title 17 U.S.C. 101 defines a United States Government work as work prepared by a military service member or employee of the United States. Government as part of that person’s official duties.

## Data availability statement

The data presented in the study are deposited in the NCBI GenBank repository and can be found at: https://www.ncbi.nlm.nih.gov/genbank/, OM281134-OM281136; OM28112; OM281162; OM281137-OM281146; OM281148 -OM281155; OM281157; OM281160-OM281168; OM281170-OM281171; OM2811173 -OM281177; OM281179-OM281185; OM281187 -OM281192; OM281156; OM281158; OM281159; OM281169; OM281178; OM281186; OM281147; OM281193-OM281217; OM320148 -OM320155; OM320157; OM281118-OM281120; OM281122 -OM281128; OM28121; OM281232; OM281229 -OM281231; OM333168-OM333184.

## Author contributions

DA: methodology, formal analysis, sample collection, investigation, and writing—original draft. AL and GM: formal analysis, data visualization, and writing—original draft. RT: methodology, formal analysis, investigation, data curation, writing—review and editing, and visualization. JS and NC: methodology, formal analysis, and investigation. BP-S, JF, and EL: conceptualization, writing—review and editing, supervision, project administration, and funding acquisition. BBd: investigation, writing—review and editing, and project administration. SD and JH: sample collection, conceptualization, and funding acquisition. BBr: morphological identification and sample collection. NT: writing—review and editing, supervision, and project administration. MF: conceptualization, formal analysis, investigation, data curation, writing—original draft, visualization, supervision, and funding acquisition. All authors contributed to the article and approved the submitted version.

## Funding

This work was supported/funded by work unit number D0016 with funding provided by the Armed Forces Health Surveillance Division (AFHSD) Global Emerging Infections Surveillance (GEIS) Branch, ProMIS ID # PO133_19_AF_N2. Lab analysis was funded by the AFHSD-GEIS under study #P0128_20_AF_14.

## Conflict of interest

The authors declare that the research was conducted in the absence of any commercial or financial relationships that could be construed as a potential conflict of interest.

## Publisher’s note

All claims expressed in this article are solely those of the authors and do not necessarily represent those of their affiliated organizations, or those of the publisher, the editors and the reviewers. Any product that may be evaluated in this article, or claim that may be made by its manufacturer, is not guaranteed or endorsed by the publisher.

## Footnotes

^1^ https://1212.mn/

^2^ https://services.arcgis.com/P3ePLMYs2RVChkJx/arcgis/rest/services/MNG_Boundaries_2018/FeatureServer

^3^ https://www.cdc.gov/westnile/resourcepages/mosqSurvSoft.html

## References

[ref1] Angelakis E. Billeter S. A. Breitschwerdt E. B. Chomel B. B. Raoult D. (2010). Potential for tick-borne Bartonelloses. Emerg. Infect. Dis. 16, 385–391. doi: 10.3201/eid1603.091685, PMID: 20202411 PMC3322042

[ref2] Aung A. K. Spelman D. W. Murray R. J. Graves S. (2014). Rickettsial infections in Southeast Asia: implications for local populace and febrile returned travelers. Am. J. Trop. Med. Hyg. 91, 451–460. doi: 10.4269/ajtmh.14-0191, PMID: 24957537 PMC4155544

[ref3] Barnes A. N. Baasandavga U. Davaasuren A. Gonchigoo B. Gray G. C. (2020). Knowledge and practices surrounding zoonotic disease among Mongolian herding households. Pastoralism 10:8. doi: 10.1186/s13570-020-00162-5

[ref4] Battsetseg B. Xuan X. Ikadai H. Bautista J. L. Byambaa B. Boldbaatar D.. (2001). Detection of *Babesia caballi* and *Babesia equi* in *Dermacentor nuttalli* adult ticks. Int. J. Parasitol. 31, 384–386. doi: 10.1016/s0020-7519(01)00120-5, PMID: 11306116

[ref5] Biggs H. M. (2016). Diagnosis and management of tickborne rickettsial diseases: Rocky Mountain spotted fever and other spotted fever group Rickettsioses, Ehrlichioses, and Anaplasmosis—United States. MMWR Recomm. Rep. 65, 1–44. doi: 10.15585/mmwr.rr6502a1 27172113

[ref6] Billeter S. A. Diniz P. P. V. P. Battisti J. M. Munderloh U. G. Breitschwerdt E. B. Levy M. G. (2009). Infection and replication of *Bartonella* species within a tick cell line. Exp. Appl. Acarol. 49, 193–208. doi: 10.1007/s10493-009-9255-1, PMID: 19242658 PMC4465226

[ref7] Boldbaatar D. Byambaa B. (2015). Bloodsucking Ticks of Mongolia. Ulaanbaatar, Mongolia.

[ref8] Boldbaatar B. Jiang R.-R. von Fricken M. E. Lkhagvatseren S. Nymadawa P. Baigalmaa B.. (2017). Distribution and molecular characteristics of *rickettsiae* found in ticks across Central Mongolia. Parasite Vector. 10:61. doi: 10.1186/s13071-017-1981-3, PMID: 28153052 PMC5289011

[ref9] Cabezas-Cruz A. Gallois M. Fontugne M. Allain E. Denoual M. Moutailler S.. (2019). Epidemiology and genetic diversity of *Anaplasma ovis* in goats in Corsica. France. Parasite Vector. 12:3. doi: 10.1186/s13071-018-3269-7, PMID: 30606253 PMC6318933

[ref10] Černý J. Buyannemekh B. Needham T. Gankhuyag G. Oyuntsetseg D. (2019). Hard ticks and tick-borne pathogens in Mongolia—a review. Ticks Tick-Borne Dis. 10:101268. doi: 10.1016/j.ttbdis.2019.101268, PMID: 31471272

[ref11] Chaorattanakawee S. Wofford R. N. Takhampunya R. Katherine Poole-Smith B. Boldbaatar B. Lkhagvatseren S.. (2022). Tracking tick-borne diseases in Mongolian livestock using next generation sequencing (NGS). Ticks Tick-Borne Dis. 13:101845. doi: 10.1016/j.ttbdis.2021.101845, PMID: 34689003 PMC8665119

[ref12] Cheslock M. A. Embers M. E. (2019). Human bartonellosis: an underappreciated public health problem? Trop. Med. Infect. Dis. 4:69. doi: 10.3390/tropicalmed4020069, PMID: 31010191 PMC6630881

[ref13] Cotté V. Bonnet S. Le Rhun D. Le Naour E. Chauvin A. Boulouis H.-J.. (2008). Transmission of *Bartonella henselae* by *Ixodes ricinus*. Emerg. Infect. Dis. 14, 1074–1080. doi: 10.3201/eid1407.071110, PMID: 18598628 PMC2600320

[ref14] Deurenberg R. H. Bathoorn E. Chlebowicz M. A. Couto N. Ferdous M. García-Cobos S.. (2017). Application of next generation sequencing in clinical microbiology and infection prevention. J. Biotechnol. 243, 16–24. doi: 10.1016/j.jbiotec.2016.12.022, PMID: 28042011

[ref15] Enkhtaivan B. Narantsatsral S. Davaasuren B. Otgonsuren D. Amgalanbaatar T. Uuganbayar E.. (2019). Molecular detection of *Anaplasma ovis* in small ruminants and ixodid ticks from Mongolia. Parasitol. Int. 69, 47–53. doi: 10.1016/j.parint.2018.11.004, PMID: 30458297

[ref16] Fischer T. Myalkhaa M. Krücken J. Battsetseg G. Batsukh Z. Baumann M. P. O.. (2020). Molecular detection of tick-borne pathogens in bovine blood and ticks from Khentii. Mongolia. Transbound Emerg. Dis. 67, 111–118. doi: 10.1111/tbed.13315, PMID: 31464102

[ref17] Gao Y. Lv X.-L. Han S.-Z. Wang W. Liu Q. Song M. (2021). First detection of *Borrelia miyamotoi* infections in ticks and humans from the northeast of Inner Mongolia. China. Acta Trop. 217:105857. doi: 10.1016/j.actatropica.2021.105857, PMID: 33582142

[ref18] He X. Zhao J. Fu S. Yao L. Gao X. Liu Y.. (2018). Complete genomic characterization of three tick-borne encephalitis viruses detected along the China-North Korea border, 2011. Vector-Borne Zoonot. 18, 554–559. doi: 10.1089/vbz.2017.2173, PMID: 29742014

[ref19] Huang T. Zhang J. Sun C. Liu Z. Haiyan H. Wu J.. (2020). A novel arthropod host of brucellosis in the arid steppe ecosystem. Front. Vet. Sci. 7:566253. doi: 10.3389/fvets.2020.566253, PMID: 33195543 PMC7649779

[ref20] Javkhlan G. Enkhtaivan B. Baigal B. Myagmarsuren P. Battur B. Battsetseg B. (2014). Natural *Anaplasma phagocytophilum* infection in ticks from a forest area of Selenge province. Mongolia. Western Pacific Surv. Resp J. 5, 21–24. doi: 10.5365/WPSAR.2013.4.3.001, PMID: 24734213 PMC3984964

[ref21] Jiao J. Lu Z. Yu Y. Ou Y. Fu M. Zhao Y.. (2021). Identification of tick-borne pathogens by metagenomic next-generation sequencing in *Dermacentor nuttalli* and *Ixodes persulcatus* in Inner Mongolia. China. Parasite Vector. 14:287. doi: 10.1186/s13071-021-04740-3, PMID: 34044867 PMC8161991

[ref23] Li H. Zhang P.-H. Huang Y. Du J. Cui N. Yang Z.-D.. (2018). Isolation and identification of *Rickettsia raoultii* in human cases: a surveillance study in 3 medical centers in China. Clin. Infect. Dis. 66, 1109–1115. doi: 10.1093/cid/cix917, PMID: 29069294

[ref24] Lkhagvatseren S. Hogan K. M. Boldbaatar B. von Fricken M. E. Anderson B. D. Pulscher L. A.. (2019). Discrepancies between self-reported tick bites and evidence of tick-borne disease exposure among nomadic Mongolian herders. Zoonoses Public Hlth. 66, 480–486. doi: 10.1111/zph.12579, PMID: 30969028 PMC6629472

[ref25] Maggi R. G. Kosoy M. Mintzer M. Breitschwerdt E. B. (2009). Isolation of *Candidatus Bartonella melophagi* from human blood. Emerg. Infect. Dis. 15, 66–68. doi: 10.3201/eid1501.081080, PMID: 19116054 PMC2660712

[ref26] Moore T. C. Pulscher L. A. Caddell L. von Fricken M. E. Anderson B. D. Gonchigoo B.. (2018). Evidence for transovarial transmission of tick-borne *rickettsiae* circulating in northern Mongolia. PLOS Neglect. Trop. Dis. 12:e0006696. doi: 10.1371/journal.pntd.0006696, PMID: 30148847 PMC6128658

[ref27] Ni J. Ren Q. Lin H. Aizezi M. Luo J. Luo Y.. (2021). Molecular evidence of *Bartonella melophagi* in ticks in border areas of Xinjiang. China. Front. Vet. Sci. 8:675457. doi: 10.3389/fvets.2021.675457, PMID: 34239911 PMC8258404

[ref28] Noguchi H. (1926). Etiology of Oroya fever. J. Exp. Med. 44, 729–734. doi: 10.1084/jem.44.5.729, PMID: 19869219 PMC2131200

[ref29] Nouchi A. Monsel G. Jaspard M. Jannic A. Angelakis E. Caumes E. (2018). *Rickettsia sibirica mongolitimonae* infection in a woman travelling from Cameroon: a case report and review of the literature. J. Travel Med. 25:tax074. doi: 10.1093/jtm/tax074 29394384

[ref30] Ochirkhuu N. Konnai S. Odbileg R. Murata S. Ohashi K. (2017). Molecular epidemiological survey and genetic characterization of *Anaplasma s*pecies in Mongolian livestock. Vector-Borne Zoonot. 17, 539–549. doi: 10.1089/vbz.2017.2111, PMID: 28678004

[ref31] Odontsetseg N. Uuganbayar D. Tserendorj S. Adiyasuren Z. (2009). Animal and human rabies in Mongolia. Rev Sci tech. OIE. 28, 995–1003. doi: 10.20506/rst.28.3.1942 20462156

[ref32] Pulscher L. A. Moore T. C. Caddell L. Sukhbaatar L. von Fricken M. E. Anderson B. D.. (2018). A cross-sectional study of small mammals for tick-borne pathogen infection in northern Mongolia. Infect. Ecol. Epidemiol. 8:1450591. doi: 10.1080/20008686.2018.1450591, PMID: 29696073 PMC5912330

[ref33] Reis C. Cote M. Le Rhun D. Lecuelle B. Levin M. L. Vayssier-Taussat M.. (2011). Vector competence of the tick *Ixodes ricinus* for transmission of *Bartonella birtlesii*. PLoS Negl. Trop. Dis. 5:e1186. doi: 10.1371/journal.pntd.0001186, PMID: 21655306 PMC3104967

[ref34] Shao J.-W. Zhang X.-L. Li W.-J. Huang H.-L. Yan J. (2020). Distribution and molecular characterization of *rickettsiae* in ticks in Harbin area of northeastern China. PLoS Negl. Trop. Dis. 14:e0008342. doi: 10.1371/journal.pntd.0008342, PMID: 32497120 PMC7272007

[ref35] Takhampunya R. Korkusol A. Pongpichit C. Yodin K. Rungrojn A. Chanarat N.. (2019). Metagenomic approach to characterizing disease epidemiology in a disease-endemic environment in northern Thailand. Front. Microbiol. 10:319. doi: 10.3389/fmicb.2019.00319, PMID: 30863381 PMC6399164

[ref36] Tokarz R. Tagliafierro T. Sameroff S. Cucura D. M. Oleynik A. Che X.. (2019). Microbiome analysis of *Ixodes scapularis* ticks from New York and Connecticut. Ticks Tick Borne Dis. 10:894–900. doi: 10.1016/j.ttbdis.2019.04.011, PMID: 31023629

[ref37] von Fricken M. E. Lkhagvatseren S. Boldbaatar B. Nymadawa P. Weppelmann T. A. Baigalmaa B.-O.. (2018). Estimated seroprevalence of *Anaplasma* spp. and spotted fever group *Rickettsia* exposure among herders and livestock in Mongolia. Acta Trop. 177, 179–185. doi: 10.1016/j.actatropica.2017.10.015, PMID: 29054570 PMC5671362

[ref38] von Fricken M. E. Qurollo B. A. Boldbaatar B. Wang Y.-W. Jiang R.-R. Lkhagvatseren S.. (2020a). Genetic diversity of *Anaplasma* and *Ehrlichia* bacteria found in *Dermacentor* and *Ixodes* ticks in Mongolia. Ticks Tick-Borne Dis. 11:101316. doi: 10.1016/j.ttbdis.2019.101316, PMID: 31677968

[ref39] von Fricken M. E. Voorhees M. A. Koehler J. W. Asbun C. Lam B. Qurollo B.. (2020b). Molecular characteristics of *Rickettsia* in ticks collected along the southern border of Mongolia. Pathogens 9:943. doi: 10.3390/pathogens9110943, PMID: 33202715 PMC7696098

[ref40] Voorhees M. A. Padilla S. L. Jamsransuren D. Koehler J. W. Delp K. L. Adiyadorj D.. (2018). Crimean-Congo hemorrhagic fever virus, Mongolia, 2013-2014. Emerg. Infect. Dis. 24, 2202–2209. doi: 10.3201/eid2412.180175, PMID: 30457521 PMC6256378

[ref41] Wechtaisong W. Bonnet S. I. Chomel B. B. Lien Y.-Y. Chuang S.-T. Tsai Y.-L. (2021). Investigation of Transovarial transmission of *Bartonella henselae* in *Rhipicephalus sanguineus* sensu lato ticks using artificial feeding. Microorganisms 9:2501. doi: 10.3390/microorganisms9122501, PMID: 34946103 PMC8705908

[ref42] Wei F. Song M. Liu H. Wang B. Wang S. Wang Z.. (2016). Molecular detection and characterization of zoonotic and veterinary pathogens in ticks from northeastern China. Front. Microbiol. 7:1913. doi: 10.3389/fmicb.2016.01913, PMID: 27965644 PMC5126052

[ref43] Wikswo M. E. Hu R. Metzger M. E. Eremeeva M. E. (2007). Detection of *Rickettsia rickettsii* and *Bartonella henselae* in *Rhipicephalus sanguineus* ticks from California. J. Med. Entomol. 44, 158–162. doi: 10.1603/0022-2585(2007)44[158:dorrab]2.0.co;2, PMID: 17294935

[ref44] Wu Q. Li J. Wang W. Zhou J. Wang D. Fan B.. (2021). Next-generation sequencing reveals four novel viruses associated with calf diarrhea. Viruses 13:1907. doi: 10.3390/v13101907, PMID: 34696337 PMC8537473

[ref45] Yin X. Guo S. Ding C. Cao M. Kawabata H. Sato K.. (2018). Spotted fever group *Rickettsiae* in Inner Mongolia, China, 2015-2016. Emerg. Infect. Dis. 24, 2105–2107. doi: 10.3201/eid2411.162094, PMID: 30334715 PMC6200000

[ref46] Yin H. Luo J. (2007). Ticks of small ruminants in China. Parasitol. Res. 101, 187–189. doi: 10.1007/s00436-007-0688-3 17823826

[ref47] Zając V. Wójcik-Fatla A. Dutkiewicz J. Szymańska J. (2015). *Bartonella henselae* in eastern Poland: The relationship between tick infection rates and the serological response of individuals occupationally exposed to tick bites. J. Vector Ecol. 40, 75–82. doi: 10.1111/jvec.12135, PMID: 26047187

[ref48] Zhang F. Liu W. Wu X.-M. Xin Z.-T. Zhao Q.-M. Yang H.. (2008). Detection of *Francisella tularensis* in ticks and identification of their genotypes using multiple-locus variable-number tandem repeat analysis. BMC Microbiol. 8:152. doi: 10.1186/1471-2180-8-152, PMID: 18798995 PMC2567983

